# Return to performance criteria in soccer after musculoskeletal injury: A scoping review

**DOI:** 10.1002/ksa.70180

**Published:** 2025-12-01

**Authors:** Peter Eppinga, Wouter Welling, Rienk Dekker, Hans Zwerver

**Affiliations:** ^1^ Center for Human Movement Sciences, University Medical Center Groningen University of Groningen Groningen The Netherlands; ^2^ MCZ Fysiotherapie en Sportbegeleiding Groningen The Netherlands; ^3^ Institute of Training Medicine and Training Physiology, TGTF, Royal Netherlands Army Utrecht The Netherlands; ^4^ Department of Rehabilitation, University Medical Center Groningen University of Groningen Groningen The Netherlands; ^5^ Sports Valley, Department of Sports Medicine Hospital Gelderse Vallei Ede The Netherlands

**Keywords:** criteria, decision making, musculoskeletal injury, return to performance, return to sport, soccer

## Abstract

Return to performance (RTPe), the final stage of the return to sport (RTS) continuum remains poorly defined in current soccer‐related musculoskeletal (MSK) research, with limited identification and application of physical and/or psychological performance‐based indicators. This scoping review aimed to systematically identify and synthesise currently used RTPe criteria, with a specific focus on facilitating return to competitive soccer at the same or higher level than pre‐injury and identify gaps in relation to sport‐specific demands. A scoping review was conducted in accordance with the PRISMA‐ScR guidelines, using comprehensive Boolean search strategies across PubMed, CINAHL, and Web of Science databases. A total of twenty‐nine peer reviewed studies met the inclusion criteria. Reported RTPe criteria encompassed the following domains: clinical examination, strength assessment, functional testing, performance‐based testing, time elapsed since injury, and patient‐reported outcome measures (PROMs). Most studies concentrated on the second stage of the three‐stage RTS continuum, that is, ‘Return to Sports’, with limited integration of on‐field, sport specific, or ecologically valid performance assessments. In addition, studies frequently neglect recommendations advocating a multidimensional and standardised test battery. Moreover, psychological readiness and performance‐level demands—critical components of RTPe in elite soccer—were seldom addressed or often lacked standardised definitions. Female athletes were underrepresented despite higher injury incidence (6.77 vs. 5.70/1000 h in males). Anterior cruciate ligament (ACL) injuries were overrepresented, whereas more frequently occurring injuries like posterior thigh and groin injuries received less attention. More than half of the studies focused on elite athletes, limiting generalisability to recreational level. These findings underscore the urgent need for more robust, operationalized, and sport‐specific RTPe criteria to support clinical decision‐making and optimise outcomes following musculoskeletal injury in soccer. Existing criteria do not reliably capture readiness to pre‐injury performance levels, addressing performance metrics, sport specific demands, and sex‐specific considerations.

**Level of Evidence:** N/A.

**Abbreviations**
GPS: Global Positioning System
MRI: magnetic resonance imaging
MSK: muskuloskeletal
RTP: return to participation
RTPe: return to performance
RTS: return to sport

## INTRODUCTION

Soccer is regarded a pivoting intermittent team sport, for which complex physical and cognitive skills are required [44, 55, 56]. The physical demands of the activity differ according to position, sex, and level of the athlete, with aerobic systems supporting the majority of activity, while key actions are predominantly performed anaerobically [7, 8, 60]. The ability to perform optimally contingent upon a combination of factors, including sprinting ability, agility, intermittent endurance, and perceptual‐cognitive skills such as anticipation and decision‐making. It is well‐documented that these abilities can undergo a decline during the process of return to sports (RTS) following an injury [2, 8, 76].

Injury risk in soccer is substantial, especially in matches where incidence is up to ten times higher than in training [38]. Injury rates in professional male and female soccer players have been documented 8.1 and 6.1 per 1000 h of play, respectively. Lower limb injuries are the most prevalent among both sexes (6.8 injuries/1000 h of exposure), with 30% lasting more than one month [34, 38, 68].

Post‐injury, rehabilitation aims to restore soccer‐specific performance and prevent reinjury [4]. However, reinjury rates remain high (14%–35%) [32, 68], indicating a need for improved RTS strategies to reduce injury burden [6].

A widely referenced model is that of Ardern who proposes a three‐stage RTS continuum [4];

1. Return to participation (RTP): The player may be participating in their sport with modification and restrictions and at a level lower than his or her RTS goal.
2. Return to sport (RTS): The player has returned to their defined sport but not yet performed at their desired level. This stage is occasionally regarded as the end‐stage for an athlete.
3. Return to performance (RTPe): The player returns their defined sport and is performing at or above the pre‐injury level.

The employment of precise terminology serves to enhance the evaluation of recovery and performance post‐injury context [19]. Doege et al. [19] define RTS as participating at the same or higher level, corresponding to Stage 3 (RTPe) of the RTS continuum.

Clinicians use evaluation tools to monitor rehabilitation, ensuring appropriate responses to treatment and readiness for progression [14]. Recent approaches emphasise objective physical and psychological outcomes over time‐based and subjective criteria [4]. The 2016 consensus statement [4] outlines the following recommendations for selecting test batteries to assess the third (RTPe) stage:

1. Using a group of tests (test battery).
2. Prioritising open tasks (less controlled) over closed tasks (more controlled) when feasible.
3. Including tests with reactive decision‐making.
4. Assessing psychological readiness to return to sport.
5. Monitoring internal (e.g., heartrate) and external (e.g., GPS data) load.

In the end‐stage of rehabilitation, evaluation tests include quantitative and qualitative measures that assess strength, function, pain, and psychological factors such as; ‘fear of reinjury’, ‘self‐efficacy’, and motivation [4, 20]. These factors do not exceed the second stage of the RTS continuum [15]. In addition, it is imperative to consider the physical performance parameters and goals for the third stage, RTPe, when engaging in the decision‐making process [12, 15, 52, 61].

The decisive factors for re‐injury associated with each stage of RTS in soccer remain to be elucidated [21, 24]. The relevance of evaluation tests in (clinical) practice remains unclear, as does which tests are clearly related to injury or re‐injury after RTS. Moreover, there appears to be a lack of knowledge surrounding the definition of the physical and/or psychological performance‐based indicators deemed pertinent for the third stage of the RTS continuum in soccer, that is, RTPe.

The objective of this scoping review was twofold: firstly, to screen existing literature and derive performance‐based criteria currently being applied for a safe RTPe in soccer after time‐loss musculoskeletal (MSK) injury; and secondly to provide recommendations for future research in this important sports medicine topic.

## MATERIALS AND METHODS

### Protocol and registration

Since the research question is of an exploratory and descriptive nature, a scoping review design and methodology was applied [5, 45, 51]. The Preferred Reporting Items for Systematic Reviews and Meta‐Analysis Extension for Scoping Reviews (PRISMA‐ScR) checklist [66] and the Population‐Intervention‐Comparators‐Outcome (PICOS) strategy were followed to conduct this scoping review. The present scoping review was registered in the Open Science Framework (OSF: DOI 10.17605/OSF.IO/GHJ9N).

### Literature search strategy

A librarian‐assisted literature search was conducted on the following electronic databases: PubMed/MEDLINE, CINAHL, and Web of Science. The databases were initially searched on 24 November 2023, and an update was performed on 24 June 2024. To ensure comprehensiveness and avoid missing any relevant studies, a final search was performed on 15 January 2025. Study inclusion ended on 1 January 2025.

### Eligible criteria

Publications were eligible for inclusion if data was reported for a research population consisting of soccer players' rehabilitation following a musculoskeletal injury toward full sports participation.

The search was limited to humans, the English language, and peer‐reviewed publications. The eligibility criteria were assured by a PICOS approach, from which inclusion in this review is based, and the following search strategy was defined:

(1) *Population*: Soccer players of any competition level, sex, and (an average) age above 18, who sustained a time loss MSK injury. At least 25% of the participants of the study needed to fit the required description above.
(2) *Intervention*: Time loss or incapacity due to MSK injury.
(3) *Comparison*: Not applicable
(4) *Outcomes*: Any criterion used for clearance to RTS, with a special interest on RTPe, mentioned.
(5) *Study designs*: Experimental and quasi‐experimental trials (e.g., randomised clinical trials, cohort studies, or cross‐sectional studies), Delphi consensus studies, surveys, and case‐control studies.

After assessing eligibility, the following exclusion criteria were applied: [1] publications in other language than English [2], systematic reviews, meta‐analyses, news articles, blogs, editorials, conference abstracts, narrative reviews [3] publications without full‐text available.

According to the search strategy studies were included for relevant publications using a Boolean phrasing of the keywords presented in Appendix A: Table A1.

### Identification and selection

Potentially relevant references from the selected electronic databases were exported to End‐note. Following de‐duplication, the references were downloaded to the Rayyan reference management platform (rayyan.ai). The first and second combined screening phases of titles and abstracts were conducted by two authors (PE & WW) independently. During title and abstract screening, if at least one reviewer, concluded that a study met the selection criteria, or if it was unclear whether the study should be included or excluded, the study was discussed until consensus was met. When the first and second authors could not reach a consensus, a third reviewer (JZ) conducted the final screening.

The reference lists of relevant systematic reviews identified during the title and abstract screen were also hand‐searched to identify potential studies that may have been missed in the electronic database search [31].

After the screening of the title and abstract, the included studies were screened in full text by the first author. The second independent reviewer (WW) scored a randomly selected 10% sample to verify the used criteria. Discrepancies or doubts after full‐text screening were resolved via consensus or discussion with the third review (JZ) for final in‐ or exclusion.

### Data extraction

From the included full‐text articles; mentioned RTPe criteria, level of play, MSK injury location, study objective, study population, as well as general demographic data were extracted. An Excel spreadsheet was used for extracting the data elements by the first author.

Additionally, a ‘RTS continuum score’ has been developed based on the five recommendations for RTS testing outlined in the consensus statement. The score ranges from 0 to 5, with 5 indicating full adherence to all the recommendations.

## RESULTS

### Study Selection

**Figure 1:**
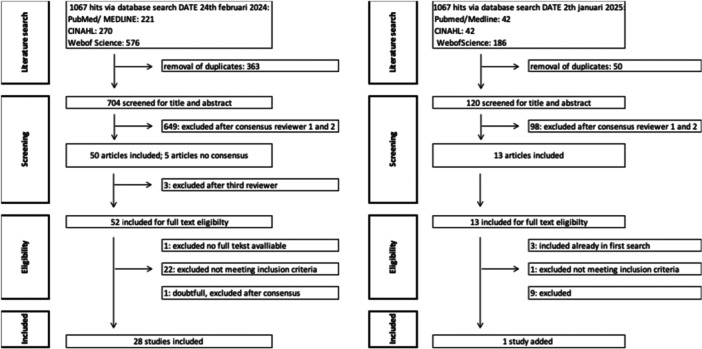
Study search flow diagram for the primary search conducted 24 June 2024 (left) and secondary (right) search conducted 15 January 2025.

The electronic database search yielded 1067 records for screening, from which 363 duplicates were removed prior to screening. After screening the titles and abstracts of 704 unique articles, 50 were selected for full‐text review. Five articles were reviewed by a third reviewer due to a lack of consensus. Ultimately, consensus between reviewers 1, 2, and 3 resulted in 28 articles meeting the eligibility criteria for inclusion in this scoping review. An update search conducted with the same framework identified one additional study. The systematic search and screening results are illustrated in a PRISMA‐ScR flowchart (Figure 1).

### Data management

After data extraction, we identified five main criteria based on consensus within the multidisciplinary research group: (1) clinical examination (including imaging diagnostics, like ultrasound and/or MRI), (2) strength, (3) functional‐assessment, (4) performance‐, (5) time and (6) patient‐reported outcomes (including pain). To highlight trends in the localisation of injury concerning used criteria, these criteria were categorised by injury location (upper versus lower extremities: ankle, knee, hip and shoulder) and injury anatomy (ligament, bone and muscle). The results of the selected studies are presented in Table 1.

**Table 1:** Summary of studies reporting on criteria used for RTPe after musculoskeletal injury in soccer.

Author (year of publication)	Main study objective	Study desing	Location (injury specification)	Sexe (number of participants) age (standard deviation (SD))	Level of play	Main criteria	Test description	Cutoff value	RTS Continuum Score (0–5)
Angelozzi et al. [3]	Investigate the rate of force development (RFD) 0 f 30,50, 90% of MVIC as an adjunct outcome measure for determing readiness for RTS following ACLR	Descriptive, prospective, longitudinal single cohort study	Lower extremity (knee‐ACLR)	Male (45)	Professional	Strength	Maximum voluntary isometric contraction/rate force development 30‐50‐90 leg press	LSI > 85%–90%	0
				Age 23,4 (SD 4,7)					
						Time	n.a.	6 months post‐operative	
Bisciotti et al. [9]	RTP after lower limb muscle injury	Consensus statement/expert opinion	Lower extremity (muscle injury)	Undefined (66)	Undefined	Clinical examination	Passive quadriceps stretch test/passive stretch leg raise test/heel raise test/squeeze test/resisted hip adduction test/pubic stress test	Not specified	3
				Age undefinied					
							Dynamic flexibility H test/ankle flexibility test/adductor passive stretching test	Not specified	
							MRI/US imaging	Not completely clear	
							Pain	Absence of	
						Strength	Muscle strength (isometric, isotonic, isokinetic) for each specific muscle function	Not specified	
						Functional assessment	Backward running/synchro plate test/drop jump test	Not specified	
						Performance	Illinois agility test/kicking test/carioca test	Not specified	
							breaking test	Not specified	
							performance evaluation	<10‐% pre injury level	
							VO2 max	90% pre injury level	
Correa et al. [17]	Compare psychological readiness and performance on physical test: field test, dynamic knee valgus, knee function, and kinesiophobia performed after ACLR	Cross sectional study	Lower extremity (knee‐ACLR)	Male ready group (15)	Competitive	Clinical examination	Knee laxity	Not specified	3
				Age 23 (SD 5.89)		Strength	Musculair strength	LSI	
				Male not ready group (20)		Functional assessment	Cross over hop test distance	LSI > 90%	
				Age 21 (SD 3.78)			Hop test	LSI	
							Knee kinematics during gait	Not specified	
							Single leg squat frontal plane projection angle	Valgus angle	
						Performance	Reactive agility test	Not specified	
							Modified Illinois change of direction	LSI	
						Patient reported	IKDC	Not specified	
							ACL‐RSI	60 points (<not ready/>ready)	
							TSK‐11	Not specified	
Dunlop et al. [21]	Return to play criteria and process in rehabilitation continuum	Survey	Lower extremity (muscle injury ‐ hamstring)	Undefined (131 teams)	Professional	Functional assessment	Non‐specified functional testing	Not specified	0
						Performance	Training load	Not specified/pre injury level?	
Fanchini et al. [23]	Description of the Return to competition after achilles tendon rupture in an elite soccer player	Case report	Lower extremity (muscle injury ‐ achilles tendon)	Male (1)	Competitive	Clinical examination	Not specified		3
							MRI	Achilles tendon thickness	
						Strength	Isokinetic assessment	LSI	
						Functional assessment	Endurance single heel raises test	5 × 25 reps	
						Performance	External training load	Total distance ‐ High intensity running distance (HIR‐distance above 16 km/u) ‐ Moderate intensity running distance (MIR‐distance between 13–16 km/u) ‐ Total distance per minute	
							Internal training load	Heartrate	
						Patient reported	Ability to cope with previous training request	No pain	
Figueroa et al. [25]	Return to Sports after anterior cruciate ligament reconstruction	Delphi study	Lower extremity (knee‐ACLR)	Undefined (34)	Professional	Clinical examination	Knee laxity	Not specified	2
							Range of motion	Not specified	
							Function	Not specified	
						Strength	Muscle strength	LSI < 10%	
						Functional assessment	Hoptesting	Movement quantity‐ and quality assessment	
						Patient reported	IKDC	Above 94,8%	
							Tegner score	Not specified	
							Psychological readiness	Not specified	
							Patient symptoms as pain‐swelling‐instability‐giving away‐locking sensation‐stifness	Not specified	
							Patient reported satisfactory	Not specified	
Forelli et al. [26]	RTS ecological situation	Descriptive	Lower extremity (knee‐ACLR)	Undefined	Undefined	Clinical examination	Range of motion	Not specified	*5*
							Craft laxity	Not specified	
						Strength	Isokinetic testing	Peak torque LSI > 90%	
						Functional assessment	Hop test: SHT/THT/SLVHT/Crossover hop	LSI > 90%, QASLS < 1	
							Coordination	Abalkov and CMJ ( < 6 cm is good coordination)	
							Plyometric	DVJ and SJ ( < 6 cm differences is not good and > 10 cm good plyometric qualities)	
							Reactive strength	DVJ and SJ (Reactive strength Index > 2,5)	
							Running assessment	Not specified	
							Vertical jump	Force Velocity Profile (F0 and limb maximum power (Pmax))	
							Postural dynamic control	Star excursion balance test < 4 cm diffrence	
								Y balance test	
							Postural control static	Centre of presure (eyes open/eyes closed × 100) > 93%	
						Performance	Game stimulation	5 min intensive period of play	
							Workload	Foster method used: 90% of the workload of the highest load of the highest session workload of the week of the training group	
							Dual task	Assess quality of movement	
							Agility test	Modified Illinois Agility Testr, AFL Agility run test/T‐tets/Reactive shuttle agility run/curve sprint test	
							Deceleration	GPS‐ measurement of intensity and braking distance	
							Fatique after game simulation	Movement analyses	
							Repeated sprint ability	6–12 efforts of 20–40 m with 30 s of recovery time	
						Patient reported	Psychological assessment (kinesiophobia, qualitty of life, fear of reinjury)	ACL‐RSI > 81.3%	
Gomez‐Piqueras et al. [30]	Use of functional performance tests in sports: Evaluation proposal for football players in the rehabilitation phase for RTS. Specify the most usefull functional test after injury	Expert opinion	Undefined	Male (16)	1st/2nd Division Spain	Functional assessment	Counter movement jump	Not specified	1
				Fitness coach (7)			Single hop test	Not specified	
				Rehab fitness coach (9)			Triple hop test	Not specified	
				Age undefinied			Crossover hoptest	Not specified	
							Star excursion balance test	<4 cm diff	
							Y balance test	Not specified	
						Performance	Yo Yo intermittent recovery for endurance	Not specified	
							Barrow test	Not specified	
							Shuttle run 8 × 5 m	Not specified	
Hgglund et al. (2007) [32]	Injuries in sport: Effect of coach control RTP intervention programme	Randomised clinical trial	Undefined	Male intervention (241)	Amateur level 6th division Sweden	Clinical examination	Effusion	Not specified	0
				Age 24 (range 14–42)		Patient reported	Pain	Not specified	
				Male control (241)					
				Age 24 (range 15–46)					
Kiani Haft Lang et al. [36]	Difference in neurocognitive function	Cross sectional study	Lower extremity (knee‐ACLR)	Male ACLR (30)	Undefinied	Strength	Quadriceps strength Index maximum voluntary contraction measured with HandHeldDynamomtry at 90 degrees hip/knee flexion	LSI 90%	1
				Age 24.93 (SD 3.78)					
				Male healthy (15)					
				Age 23.29 (SD 1.49)					
						Functional assessment	Single leg hop for distance	LSI 90%	
							Tripple hop for distance	LSI 90%	
							Tripple cross over hop for distance	LSI 90%	
							6‐m timed hop test	LSI 90%	
						Patient reported	Knee Outcome Survey Activity of Daily living Scale	Not specified	
							Global Knee Rating Scale	Not specified	
Kurz et al. [37]	Prospective value of RTS assessment after shoulder injury	Explorative prospective multicentre cohort study	Upper extremity (shoulder)	Undefined	Undefined	Clinical examination	range of motion	TROM (within 5° right‐left)	2
				Age 18–35			Upper limb strength	IR (degree)	
								ER (degree)	
							Antropometry (Height/Weight)	Not specified	
						Strength	Isolated and complex function		
							Handgrip strength: absolute and relative strength. testing differences injured versus noninjured side	Not specified	
							Isometric strength PeakTorque 90 degrees Abduction, 0 and 90 degrees ExternalRotation. testing differences injured versus noninjured side	Not specified	
							Isometric strength PeakTorque 90 degrees Abduction, 0 and 90 degrees InternalRotation. testing differences injured versus noninjured side	Not specified	
							Isometric strength PeakForce eccentric 30 and 90 degrees Abduction. testing differences injured versus noninjured side	Not specified	
							External rotation:Internal rotation ratio	Not specified	
							Eccentric‐Internal rotation ratio	Not specified	
							External rotation‐abduction ratio	Not specified	
							Isokinetic external‐/internal‐rotation testing (mean and peak torque and ratio)/testing differences injured versus noninjured side	Not specified	
							Abduction adduction testing supine position. testing differences injured versus noninjured side	Not specified	
						Functional assessment	Upper quarter Y‐balance test (UQYBT). testing differences injured versus noninjured side	Not specified	
							Closed kinetic chain upper extremity stability test (CKCUEST)	Not specified	
							Wall Hop test (WHT). testing differences injured versus noninjured side	Not specified	
							Functional throwing performance index (FTPI). testing differences injured versus noninjured side	Not specified	
							Unilateral seated shot put test (SSPT). testing differences injured versus noninjured side	Not specified	
						Patient reported	Western Ontario Shoulder Instability Index (WOSI): Otley modified score (100‐ ((0‐2100)/21))	>95 is clearence for RTS	
							Quick‐disabilities of the Arm, Shoulder and Hand (QuickDASH)	Not specified	
							Psychological readiness of injured athlete to return to sport (PRIA‐RS)	>40 RTS criterium	
							Shoulder instability ‐ return to sport after injury (SIRSI)	Not specified	
Maestroni et al. [38]	Study for the usage of the TSA score to prove better RTS prediction than absolute alone standing scores	Cross sectional study	Lower extremity (knee‐ACLR)	Male (95)	Elite (Qatar)	Strenght	Isokinetic knee extension and flexion strength relalive to bodyweight (isokinetic dynamometer 60 degrees/sec)	>90% LSI	1
				ACLR (60)		Functional assessment	Hop tests	>90% LSI	
				Age 25.1 (SD 12.6)			Total score of Athleticism (z‐score from 2 different tests/4 different z‐scores))	Not specified	
				Uninjured (35)					
				Age 23.8 (SD 2.8)			Countermovement jump (bilateral/unilateral): jump height/relative peak power/RSImod (jump height/contractiontime from movement onset lift off)	Not specified	
Mayer et al. [41]	Compare the performance on 2 functional tests (Upper Quaretr Y Balance Test (YBT‐LQ) and Functional Movement Scores (FMS) of ACL reconstruction after clearance to unrestricted sport participation	Cohort study	Lower extremity (knee‐ACLR)	Undefinied (98 of which 34 soccer)	Undefined	Clinical examination	Effusion	No effusion	1
				Cleared 25.6 (SD 13.2)			Range of motion	Within 5° of controlateral side	
				Not cleared 21.0 (SD (7.4)			KT‐1000 arthrometer.	<5 mm graft of the controlateral limb	
							Lachman test.	<5 mm translation	
							Pivot shift test	Negative	
						Strength	Quadriceps strength	None or mild strength deficits	
						Functional assessment	Single leg hop test for distance	Not specified	
							Crossover hoptest	Not specified	
							Tripple hoptest	Not specified	
							Timed hop test	Not specified	
						Time	Time since surgery	> 6 months post operative	
						Patient reported	Knee injury and Osteoartritis Outcome Score	Not specified	
							Knee Self Efficacy Score	Not specified	
Mendiguchia et al. [42]	Study aimed to assess the concurrent effectiveness of administering an individualised and multifactorial criteria‐based algorithm (rehabilitation algorithm [RA]) on hamstring injury rehabilitation in comparison with using a general	Double‐blind randomised controlled trial	Lower extremity (muscle injury ‐ hamstring)	Male (48)	Semi‐/professional	Clinical examination	Torsion capabilities: Active Straight Leg Raise test	No compensation	1
				Rehabilitation Algorithm group (24)					
				24 (SD 4.4)			Insecurity and Pain with Askling H‐test	No pain and insecurity	
				Rehabilitation Protocol group (24)					
				22.9 (SD 6)		Strength	isokinetic knee flexion/extension at 60 degrees/sec: Peak torque (H/H) and conventional H/Q ratio	<10% H/H, H/Q > 0.45 (biodex) or > 0.47 (Cybex)	
							Hip extension in prone position	<10% asymmetry between legs	
						Functional assessment	Triple hop for distance	<10% asymmetry between legs	
							Endurance (repition number): single leg hamstring bridge test (SLHBT)	>25 reps, < 10‐% asymmetry between legs	
						Patient reported	Pain on palpation	Not present	
Mitchell et al. [43]	Case description pathway RTS after ACL reconstruction with medial meniscectomy during the course of rehabilitation	Case report	Lower extremity (knee‐ACLR)	Male (1)	Professional	Clinical examination	Pain/effusion	Absence of	3
				Age 26		Strength	Isokinetic strength testing (90‐180‐300°/s)	Decsriptive LSI > 90%	
						Functional assessment	Single leg hop	Decsriptive LSI > 90%	
							Triple leg hop	Decsriptive LSI > 90%	
							Cross over hop	Decsriptive LSI > 90%	
						Performance	On‐field specific assessment	2 weeks of full participation to build up in game minutes	
							External load (GPS)	Not specified	
						Patient reported	Selfrated readiness	>90%	
Norouzi et al. [46]	Lower extremity kinematic analysis in 1 jump landing task and its association with return to sport criteria	Cross sectional study	Lower extremity (knee‐ACLR)	Male (27)	Local footbal clubs	Clinical examination	Range of motion	Full	1
				23 (SD 3.3)			Effusion	Trace or zero	
							pain	None	
						Strength	Maximum isometric quadriceps strength with handhelddynamometry	LSI > 0.9	
						Functional assessment	Single leg hop for distance	LSI > 0.9	
							Crossed over hop test for distance	LSI > 0.9	
							6‐m timed hop test	LSI > 0.9	
							Triple leg hop test for distance	LSI > 0.9	
						Time	Time post surgery	>6 months post surgery	
						Patient reported	KOS‐ADL	Score > 90	
							*Global Knee Rating Scale Questionnaire*	Score > 90	
Oleksy et al. [47]	The aim was to analyse how many tests to included in the RTS test battery and which tests are most indicative for functional deficits related to ACL reconstruction	Comprehensive assesment	Lower extremity (knee‐ACLR)	Male (65)	Regional	Strength	Isokinetic test protocol knee flexion‐extension at 60‐180‐300 degr/sec	LSI > 90%	1
				Age 18–25			H/Q ratio with isokinetic testing	Male < 62.5%//female < 55%	
						Functional assessment	Single hop for distance	LSI > 90%	
							Single hop for distance with biomechanical analysis	No valgus loading/altered postural stability	
Oleksy et al. [48]	The purpose was to determine whether players who have passed RTS assessment still have deficits in movement patterns or in neuromuscular control after such serious injury as ACL rupture and reconstruction	Comprehensive assesment	Lower extremity (knee‐ACLR)	Male (65)	Regional	Clinical examination	Standard orthopaedic test	Not specified	1
				Age 18–25			Manual test	Not specified	
						Strength	Isokinetic test protocol knee flexion‐extension at 60‐180‐300°/s	LSI > 90%	
							H/Q ratio with isokinetic testing	Male < 62.5%//female < 55%	
						Functional assessment	Single hop for distance	LSI > 90%	
							Single hop for distance with biomechanical analysis	No valgus loading/altered postural stability	
Pasqualini et al. [50]	Outcome after artroscopic bankart repair shoulder	Retrospective comparative cohort study		Male (81)	Recreational/competitive	Clinical examination	Range of motion	No pain	0
				Age 23–27			Apprehension test	No apprehension	
						Strength	Not specified	Same as pre ‐ operative	
Smith et al. [58]	The aim is to reach consensus on RTS decission making after an lateral ankle sprain	3 round Delphi survey approach	Lower extremity (ankle)	Health porfessionals both sexes (155)	Elite	Clinical examination	Range of motion	Not specified	4
							Effusion		
				Age 41.3 (SD 8.7)		Strength	Muscle strength	Not specified	
							Muscle endurance	Not specified	
							Muscle power	Not specified	
						Functionall assessment	Proprioception; dynamic postural control/balance	Not specified	
							Hoptestting	Not specified	
						Performance	Jumping and agility	Not specified	
							Sport specific drills	Not specified	
							Ability for full training session	Not specified	
						Patient reported	Perceived ankle confidence/reassurance and stability	Not specified	
							Psychological readiness	Not specified	
							Numeric rating scale	Not specified	
Thomson et al. [64]	Running biomechanics in ACL reconstructed athletes	Case–control study	Lower extremity (knee‐ACLR)	Male (32)	Professional or high level recreational	Clinical examination	Pain	Complete absence	4
				Age 26–28			Range of motion	Full range of motion	
							Joint examination (pivot shift/Lachman/Laxity evaluation)	Stable knee	
							Effusion	Complete absence	
						Strength	Isokinetic strength test flexion‐extension 60 degrees per second	100% symmetry/restore pre‐operative values or normative values for sports and level of activity	
						Functional assessment	Counter movement Jump	>90% symmetry of jump height and concentric and eccentric impulse	
							Drop jump	>90% symmetry of jump height and concentric and eccentric impulse	
							Reactive strength Index	>1.3 for double leg and 0.5 singe leg	
							Jumping biomechanics	Normalise absolute and symmetry values for moments, angles and work in vertical and horzontal jumps in saggital and frontal plane at hip, knee and ankle	
							Running mechanics	>90% symmetry vertical ground reaction forces and knee biomechanics during stance during high‐ speed running and change of direction	
						Performance	Complete a sport specific training programme	Not specified	
						Patient reported	IKDC	Normalised	
							ACL‐RSI	Normalised	
							Tampa Scale of Kinesiophobia	Normalised	
Tol et al. [65]	Study the effect of platelet‐rich plasma in hamstring injuries	Randomised clinical trial	Lower extremity (muscle‐hamstring)	Male (52)	Professional	Clinical examination	Range of motion hamstring	>75% uninvolved side	4
				Age 24.9 (18–38)			Range of motion straight leg raise	>75% uninvolved side	
							Flexor length	Full ROM knee extension supine	
						Functionall assessment	Humps	Painless completion	
						Performance	Field testing:directional changes	No observational limitations and or symptoms (pain)	
							Field testing: sprints	100% running speed	
							Field testing: (cross‐)passes	Painless completion	
							Field testing: shooting	Painless completion	
							Field testing: interval running	Painless completion	
							Field tetsing: one‐on‐one attacking and defence skills	Painless completion	
Valente et al. [67]	Study to describe preceptions and practices on the management of athletes with hamstring strain injuries among physical therapists	Cross sectional online survey	Lower extremity (muscle‐hamstring)	Undefined (95)	Serie A, Serie B Brazilian men's football championship	Clinical examination	Stretching	Absence of pain	2
				Age 38 (SD 6.8)			Strength	Absence of pain	
							Range of motion	Not specified	
							Imaging exam	Not specified	
						Strength	Muscle strength levels	Not specified	
						Functionall assessment	SLHB/H‐test/hop test	Not specified	
						Performance	External load	Not specified	
							Intense actions (eg accelerations)	Not specified	
							Performance in sprints/high‐speed running	Not specified	
						Time	Time since injury event	Not specified	
						Patient reported	Pain	Absence of	
van der Horst et al. [69]	RTS criteria consensus	Cross sectional Delphi study	Lower extremity (muscle‐hamstring)	Undefined	Undefined	Clinical examination	Functional testing	Absence of pain	4
							Stretching	Absence of pain	
							Manual strength testing	Absence of pain	
							Palpation	Absence of pain	
							Active and passive straight leg raise	Similar injured versus non‐injured	
						Strength	Eccentric strength	Similar injured versus non‐injured	
						Functionall assessment	Single leg bridge	Not specified	
						Performance	Repeated sprint ability test (RSA) pitch	Not specified	
							Deceleration drills on field	Not specified	
							Positione specific GPS targeted match specific rehabilitaion	Not specified	
						Patient reported'	Psychological readiness/athlete's confidence	Not specified/future research	
van Dyk et al. [70]	Isokinetic strength measurements at preseason and at RTP after hamstring injury	Randomised clinical trial	Lower extremity (muscle‐hamstring)	Male (41)	Professional (Qatar)	Clinical examination	Clinical examination sports medicin physician	Not specified	0
				Age 25 (SD 4)		Patient reported	Comments regarding on‐field sessions	Not specified	
van Melick et al. [71]	Fatique influence	Cross sectional case control study	Lower extremity (knee‐ACLR)	Male (33)	Recreational	Functionall assessment	Single leg vertical drop jump	>90% LSI	1
				Age 18–30			Single leg hop for distance	>90% LSI	
							Single leg side hop	>90% LSI	
							Double leg counter movement jump	<6	
Vergani et al. [73]	Delphi study among experts to investigate RTP criteria for long standing adductor related groin pain (LSARGP) used by each expert	3‐round Delphi study	Lower extremity (muscle‐groin)	Undefined (32)	Undefined	Strength	Hip adductor isometric squeese test	Side‐to‐side symmetry	4
							Hip adductor eccentric strength	Side‐to‐side symmetry	
						Functional assessment	Intersegmental control: single leg squat movement quality	Not specified	
						Performance	Planned‐unplanned CoD to varying degrees (45‐90‐110‐180°): T‐test + Illinois test	Absence of pain and full confidence	
						Patient reported	Copenhagen Hip and Groin Outcome Score (HAGOS)	Not specfied	
Zambaldi et al. [77]	Achieve expert consensus on RTP after hamstring injury	3‐round Delphi study	Lower extremity (muscle‐hamstring)	Undefined (18)	Undefined	Clinical examination	Passive straight leg raise flexibility	Not specified	3
							Pain	Absence of	
						Strength	Isokinetic strength (ecc)	No strength imbalances	
						Functional assessment	Lumbo‐pelvic control	Not specified	
						Performance	Max sprint/max lineair velocity	Not specified	
							High speed running	Equivalent to match requirements	
							Full aerobic and anaerobic fitness	Match based targets external load	
						Patient reported	Players confidence and feeling ready	Needs to be there	
Estevez Rodriques et al. [22]	Case description pathway RTS after adductor strain	Case report	Lower extremity (muscle‐groin)	Male (1)	Professioanl	Clinical examination	Ultrasound	Correct alligment of muscle fibres without evidence of oedema	2
				Age 26					
						Performance	External load with GPS	>75% game load	
								>90% of maximum speed	
								Accumulative sprints to RTT demands	
						Patient reported	Pain	Visual Analoque Scale (VAS) < 2	

Abbreviations: ACLR, anterior cruciate ligament reconstruction; ACL‐RSI, anterior cruciate ligament‐return to sport; CMJ, counter movement jump; CoD, change of direction; DVJ, drop vertical jump; GPS, global positioning system; H, hamstring; H test, dynamic hamstring flexibility test; IKDC, International Knee Documentation Committee; KOS ADL, Knee Outcome Survey Activity Daily Living; MVIC, maximum voluntary isometric contraction; Q, quadriceps; QASLS, qualitative analyse single leg loading; RTP, return to play; RTPe, return to performance; RTS, return to sports; RTT, return to training; SHT, single hop test; SJ, squat jump; SLVHT, single leg vertical hop test; THT, triple hop test; TSK 11, Tampa scale 11.

The study design of included studies varied in nine expert opinions (Delphi, consensus statement, and survey studies) [9, 21, 25, 30, 58, 67, 69, 73, 77], three case reports [23, 43, 78], six cross‐sectional studies [17, 36, 39, 46, 64, 67, 71], four randomised clinical trials [33, 42, 65, 70], one descriptive study [26], four cohort studies [3, 37, 41, 50] and two comprehensive assessments [47, 48].

A total of 1.165 participants and 458 expert opinions were included in this scoping review. With respect to sex, the reviewed studies included 18 studies on males only, and 11 undefined. Participants' ages ranged from 14 to 46 years with a mean age of 25, and the level of participation varied from amateur to elite.

The included studies on MSK injuries primarily focused on the lower extremities: 13 on knee injuries (predominantly anterior cruciate ligament), one on ankle ligament injuries, two on groin injuries, eight on muscle injuries (Groin/Hamstring/Achilles tendon) and three on non‐specified injuries. Upper extremity criteria were discussed in two of the studies.

### Criteria

#### ‘RTS continuum score’

Among the included studies 23 used a group of tests for assessment criteria. Nine prioritised open tasks over less controlled assessment, six incorporated reactive decision‐making testing, 10 assessed psychological readiness, and eight included measures of external and/or internal load. Only one study addressed all recommendations, achieving the maximum score of 5, while the average score across all studies was 2. Individual study scores are detailed in Appendix A: Table A2.

#### Clinical examination

Out of 29 included studies, 21 report using at least one method of clinical examination. Range of motion (ROM) and flexibility are mentioned in 13 studies. Laxity or stability testing of the joint are mentioned in 13 studies.

Imaging is utilised as a criterion in four studies; one was not specified and three used MRI and/or ultrasound. The other metrics mentioned include swelling (six studies), manual testing of strength and/or function (eight studies), anthropometric measurements (one study), palpation (one study) and pain (six studies). Additionally, one study mentioned clinical examination without further specification.

#### Strength test

Twenty‐two of the included studies use strength as a criterion, with a range of specifications on which tests to use and the criteria to objectively determine RTS. Isokinetic, isometric, and/or isotonic testing is used in upper and lower extremity testing and mentioned in 13 of the included studies. Ten studies did not specify muscle strength testing. The strength index, strength endurance, strength power, and eccentric strength are other mentioned criteria in five studies.

#### Functional assessment

Twenty‐four studies report functional assessment caried out in laboratory‐like settings. Four studies address the assessment of walking and/or running, mentioning gait analysis during walking or running and evaluating the possibility of backward running. Four studies mention testing single‐leg function, examining the ability to perform a bridge or squat, and one study conducted the heel raise test. Hop tests are mentioned in 19 studies, with five studies mentioning the crossover hop test and 14 using a battery of hop tests. Both the Strength Index and the Reactive Strength Index are mentioned in two studies. Postural control is assessed in four studies: two use dynamic assessments, and two use static assessments. Other functional tests mentioned include the Albakov test (three studies), Y balance test (three studies), star excursion test (one study), Wall Hop Test (WHT) (one study), Closed Kinetic Quality Upper Extremity Strength Test (CKQUEST) (one study), and the Unilateral Seated Shot‐Put Test (USSPT) (one study).

#### Performance assessment

Performance assessment is mentioned as a criterion in 15 studies. Criteria specified outcome is often ‘absence of pain’ or ‘ability to perform’. Some studies use the limb symmetry index as an objective criterion. Outcome measurements for soccer are not specified except GPS load variables which are related to percentage of pre ‐injury levels.

### Time to RTS

Time is reported as a criterion in three of the 29 included studies. Two studies reported a time frame for clearance after surgery, while one study did not specify the time after injury.

### Patient‐reported outcomes

Patient‐reported outcome criteria are utilised in 19 of the 29 included studies. These criteria included subcategories such as patient‐reported function, psychological readiness and pain. Pain, measured with numeric rating scale or visual analogue scale, is specifically described as an outcome measure in two studies. The absence of pain is reported as a criterion within clinical assessment and functional or performance testing in six studies. Eleven studies include psychological readiness as a criterion.

## DISCUSSION

This scoping review aimed to provide an overview of the current criteria for a safe and successful RTPe used in soccer after a time‐loss MSK injury. Reported criteria covered the domains of clinical examination, strength, functional‐ and performance‐testing, time, and patient‐reported outcome (absence of pain and psychological readiness). However, the 29 studies reviewed primarily focus on RTS rather than RTPe. They often lack sport‐specific tests that reflect the physical, physiological, and psychological demands required to compete at the pre‐injury level or higher. Most commonly, criteria involve injury‐specific assessments in controlled lab environments. Additionally, contextual and participation‐related factors across the RTS continuum are rarely addressed, and research on post‐injury athletic performance remains limited.

Despite being an emerging sport for females with a relatively high incidence of MSK injuries, with an overall incidence of 6.77 injuries per 1000 hours in females versus 5.70 injuries per 1000 h in males, surprisingly, none of the included studies focused exclusively on female participants. Females predominantly sustain joint and ligament injuries to the knee and ankle, whereas males are more likely to suffer thigh muscle injuries [57]. The lack of research on female soccer players is concerning, as growing evidence suggests distinctly different injury patterns between male and female athletes. Further investigation is particularly needed to address these sex‐specific injury characteristics and their impact on RTS in female soccer players [40].

Also, the level of play among participants in the included studies is of interest. More than half of the included studies focused on elite or professional athletes. This focus may be due to the availability of time, data, and rehabilitation and testing resources at these levels. However, it raises the question if these results can be extrapolated and how applicable these criteria are in non‐elite and recreational athletes. Future RTS and RTPe research should include the latter groups to reach broader applicability.

A third remark pertains to the types of MSK injuries discussed. The studies reported injuries to the muscles (groin and hamstring) and joints (knee, ankle, and shoulder), with knee injuries being the most frequently addressed. The primary injury discussed (half of the included studies) was the anterior cruciate ligament (ACL). However, evidence shows that hip and groin injuries (1.35/1000 h) and posterior thigh muscle injuries (1.83/1000 h) have higher incidence rates compared to knee injuries (0.7/1000 h) [35]. This discrepancy may be explained by the significant impact a knee ligament injury has on a player's performance and the months of time lost. Other MSK injuries are under‐investigated as well and are therefore not represented in current stages of the RTS continuum. So, there is an opportunity for advancing RTPe criteria development in these injuries. Given the heterogeneity of included MSK injuries, the diversity in applied criteria across studies is to be expected; however, some consistency can be observed (e.g., the frequent use of strength and functional testing for RTS, versus less sport‐specific performance metrics and psychological readiness assessments for RTPe).

### Criteria

Several of the included studies, specifically those utilising survey and Delphi methodologies, aimed to establish consensus on RTS criteria for pivoting sports, including soccer. However, most of these studies explored new criteria rather than achieving consensus on a unified set of criteria. This underscores the ongoing lack of consensus on RTS, with focus on RTPe (Stage 3 of the RTS continuum) standards within soccer, emphasising the importance of continued research and development of evidence‐based, safe RTS protocols in musculoskeletal rehabilitation.

The primary criteria identified in this review are clinical examination, functional‐, and performance testing, patient‐reported outcome, time and strength. These criteria serve as clinical guidelines for the rehabilitation process [15, 74]. These criteria primarily focus on identifying impairments, lacking crucial insights into the athlete's ability to manage both the physical and psychological demands in an ecological environment for RTPe. Additionally, only one study included all five recommendations for a testing battery from the consensus statement.

Performance testing is mentioned in fourteen studies included in this scoping review. On‐field performance testing is addressed, but mostly in terms of ‘conducted without pain’ or simply ‘able to perform’ lacking objective criteria and a clear description of methods or specific tests. Prioritising more on‐field testing with clear criteria for RTPe over traditional clinical assessments is necessary [12, 13, 76].

### Perspective

Our scoping review indicates that recent research in the MSK domain within soccer does not fully align with the RTS consensus statement and that performance‐based indicators for RTPe are lacking. Considering the low RTS Continuum scores, developing a more effective test battery for RTPe may lead to improvements.

Although the current focus in literature is on utilising multiple criteria to assess function, strength, and patient‐reported outcomes, performance indicators remain undefined and lack specific cut‐off values. Moreover, while the parameters employed seem conditional and provide guidance for the RTS‐continuum, their predictive value is low [15, 75]. Furthermore they fail to address the challenges associated with the later stages of physical [12, 49] and neurocognitive [53] demands for RTPe. Current test batteries are primarily clinical findings that consists of constrained closed‐skill tests in a predictable environment, whereas most MSK (re)injuries occur in unconstrained, open, and random environments. This raises a critical question: is a clinical, non‐fatigued, pre‐planned situation the most optimal method for detecting altered movement patterns [28]? Functional testing also seems to be influenced by sport‐specific loads and is influenced by sport‐specific activities [16]. Therefore, single‐point‐in‐time measurements may be less informative. For instance, the impact of gameplay on functional testing (such as hop testing) revealed a decline in performance on the previously injured side [72]. During the final phase of rehabilitation, athletes should focus on regaining soccer‐specific movements and on improving physical, technical, tactical, and psychological readiness while carefully managing internal and external workloads from controlled to increasingly dynamic environments [1, 11, 12, 61]. End‐stage assessment should take place in ecologically valid (on‐field) settings [18] with an emphasis on sport‐specific physiological demands [12]. Future research should focus more on on‐field performance testing in an ecologically valid setting.

The gradual workload progression is notably absent as a criterion. Managing the time‐course of injury risk following RTS is critical, as shown in a recent study by Zhang et al. [78], which highlighted the 'one‐month excess risk decay'. This study revealed a sharp decline in injury risk within the first four weeks after RTS for severe injuries, likely due to increased resilience from increased chronic loading [10, 27, 59, 62, 63]. However, this hypothesis warrants further investigation on its effect on RTS.

In general, physical performance and psychological readiness—not merely the absence of pain but lack of anxiety—should be a key consideration in the RTS decision‐making process [1, 12, 29, 61]. To ensure athletes return to their previous or higher levels of competition, the actual in‐game demands of men and women of various ages and competition levels should be considered [52, 62]. However, specific criteria, metrics, and thresholds remain unclear [21]. This gap in knowledge between the criteria used and RTPe supports our initial hypothesis that there is insufficient understanding of how to define the physical or psychological thresholds for RTPe in soccer, especially in relation to performance‐based indicators highlighted in this scoping review.

### Limitations and strengths

This scoping review has some strengths and limitations, which carry implications for athletes, practitioners, and researchers. A potential publication bias may exist, and some relevant tests and data could have been missed, as only peer‐reviewed studies in English were included. Additionally, unpublished or innovative assessments used in practice may not be reflected. For many of the identified tests, it remains unclear whether they are linked to a reduced risk of re‐injury, due to a lack of conceptual frameworks or evidence‐based links to RTS outcome.

On the strength side, the review utilised three databases, covering a wide range of soccer‐related injuries, providing a comprehensive overview of current RTS criteria. A scoping review, due to its broad scope, enables the mapping of existing literature and the identification of knowledge gaps, in contrast of a systematic review which typically focuses on a narrowly defined research question [54]. Conducting a scoping review allows to also include expert opinions from Delphi studies, thus expanding perspective on RTS strategies. The included studies reflect input from both the medical (physicians and physical therapists) and the performance (performance coaches and (athletic‐) trainers) domains, highlighting interdisciplinary collaboration. This overlap may foster to safer and more effective RTS decisions. Finally, the methodology employed adheres to generally accepted standards.

## CONCLUSION

Performance‐based measures and the physical and psychological demands essential for RTPe in soccer are rarely reported and often lack clear definitions, highlighting the need for further research in this area.

## RECOMMENDATIONS

Based on this scoping review we suggest that future research could focus on different areas.

First, research should aim to identify the physical requirements necessary for soccer players to perform at their level of play. Specifically, until now it remains unclear which physical and performance criteria should be met corresponding the fifth recommendation, load monitoring, of the RTS continuum for test battery development. Additionally, the physical demands of the weekly micro‐cycle and gameplay should be examined in relation to soccer. Adopting a more holistic approach to RTPe in soccer, with tailored progressions based on real‐time data ensures that players are not only medically cleared to return but are also match‐fit, resilient, and ready for the competitive demands of the sport.

Second, research should also investigate the specific demands for female soccer players, particularly the differences in physical demands between men's and women's soccer. Female soccer players may have different performance goals compared to male players, and the differences in physical abilities between male and female athletes could influence RTPe criteria and decisions.

Finally, different levels of competition may entail variations in performance demands. Further research is warranted to elucidate these distinctions and their implications for RTPe protocols, especially in contexts where monitoring resources are more limited than those available to elite soccer players.

## AUTHOR CONTRIBUTIONS

All authors conceived the review, developed the methodology and discussed the results. Peter Eppinga and Wouter Welling explored the literature and J.Z. was the third reviewer. Peter Eppinga drafted the manuscript, which all authors critically reviewed and approved for publication. All authors substantially contributed to the study's conception and are collectively responsible for the integrity and accuracy of the work.

## CONFLICT OF INTEREST STATEMENT

The authors declare no conflict of interest.

## ETHICS STATEMENT

None declared.

## Data Availability

All data used to support the findings of this study are available upon request p.eppinga01@umcg.nl.

## 1

**Table A1:** Search strategy.

Database	Search string
Pubmed	(“Soccer”[Mesh] OR soccer*[tiab] OR footbal*[tiab]) AND (“Return to Sport”[Mesh] OR “return to sport*“[tiab] OR “return to play*“[tiab] OR “sport contin*“[tiab] OR “return to competition*“[tiab] OR “return to performan*“[tiab]) AND (“Wounds and Injuries”[Mesh] OR “Postoperative Care”[Mesh] OR injur*[tiab] OR “postoperative care”[tiab] OR “post‐operative care”[tiab]) AND (“Decision Making”[Mesh] OR deci*[tiab] OR criteria*[tiab] OR guideline*[tiab]) NOT (“ brain concussion”[Mesh]) NOT (“Review” [Publication Type] OR “Meta‐Analysis” [Publication Type] OR “Systematic Review” [Publication Type] OR “systematic review”[ti])
CINAHL	((MH “Soccer”) OR TI (soccer* OR footbal*) OR AB (soccer* OR footbal*)) AND ((MH “Wounds and Injuries + “) OR TI (injur* OR “postoperative care” OR “post‐operative care”) OR AB (injur* OR “postoperative care” OR “post‐operative care”)) AND ((MH “Sports Re‐Entry”) OR TI (“Return to Sport*“ OR “return to play*“ OR “sport contin*“ OR “return to competition*“ OR “return to performan*“) OR AB (“Return to Sport*“ OR “return to play*“ OR “sport contin*“ OR “return to competition*“ OR “return to performan*“)) AND ((MH “Decision Making + “) OR TI (deci* OR criteria* OR guideline*) OR AB (deci* OR criteria* OR guideline*))
Web of Science	TS= (soccer* OR footbal*) AND TS= (injur* OR “postoperative care” OR “post‐operative care”) AND TS= (“Return to Sport*“ OR “return to play*“ OR “sport contin*“ OR “return to competition*“ OR “return to performan*“) AND TS= (deci* OR criteria* OR guideline*)

**Table A2:** RTS continuum score per included study (* average score).

Study	Using a group of tests	Prioritising open task (less controlled) over closed tasks (more controlled) when feasible	Including test with reactive decision‐making	Assessing psychological readiness to return‐to‐sport	Monitoring internal and external load	RTS continuum score
Angelozzi et al. [3]	X	X	X	X	X	0
Bisciotti et al. [9]	V	V	X	X	V	3
Correa et al. [17]	V	X	V	V	X	3
Dunlop et al. [21]	X	X	X	X	V	0
Fanchini et al. [23]	V	V	X	X	V	3
Figueroa et al. [25]	V	X	X	V	X	2
Forelli et al. [26]	V	V	V	V	V	5
Gomez‐Piqueras et al. [30]	V	X	X	X	X	1
Hägglund et al. [33]	X	X	X	X	X	0
Kiani Haft Lang et al. [36]	V	X	X	X	X	1
Kurz et al. [37]	V	X	X	V	X	2
Meastroni et al. [39]	V	X	X	X	X	1
Mayer et al. [41]	V	X	X	X	X	1
Mendiguchia et al. [42]	V	X	X	X	X	1
Mitchell et al. [43]	V	X	X	V	V	3
Norouzi et al. [46]	V	X	X	X	X	1
Oleksy et al. [47]	V	X	X	X	X	1
Oleksy et al. [48]	V	X	X	X	X	1
Pasqualini et al. [50]	X	X	X	X	X	0
Smith et al. [58]	V	V	V	V	X	4
Thomson et al. [64]	V	V	V	V	X	4
Tol et al. [65]	V	V	V	X	X	3
Valente et al. [67]	V	X	V	X	X	2
van der Horst et al. [69]	V	V	X	V	X	3
van Dyk et al. [70]	X	X	X	X	X	0
van Melick et al. [71]	V	X	X	X	X	1
Vergani et al. [73]	V	V	X	V	V	4
Zambaldi et al. [77]	V	X	X	V	V	3
Estevez Rodriques et al. [22]	X	V	X	X	V	2
**Total number of studies**	**23**	**9**	**6**	**10**	**8**	**2***
